# Reference Charts for Fetal Cerebellar Vermis Height: A Prospective Cross-Sectional Study of 10605 Fetuses

**DOI:** 10.1371/journal.pone.0147528

**Published:** 2016-01-26

**Authors:** Pietro Cignini, Maurizio Giorlandino, Pierpaolo Brutti, Lucia Mangiafico, Alessia Aloisi, Claudio Giorlandino

**Affiliations:** 1 Department of Prenatal Diagnosis, ALTAMEDICA Fetal-Maternal Medical Centre, Rome, Italy; 2 Department of Statistics, Sapienza University of Rome, Rome, Italy; 3 Department of Obstetrics and Gynecology, Campus Biomedico University of Rome, Rome, Italy; Hospital de Especialidades del Niño y la Mujer de Queretaro, MEXICO

## Abstract

**Objective:**

To establish reference charts for fetal cerebellar vermis height in an unselected population.

**Methods:**

A prospective cross-sectional study between September 2009 and December 2014 was carried out at ALTAMEDICA Fetal–Maternal Medical Centre, Rome, Italy. Of 25203 fetal biometric measurements, 12167 (48%) measurements of the cerebellar vermis were available. After excluding 1562 (12.8%) measurements, a total of 10605 (87.2%) fetuses were considered and analyzed once only. Parametric and nonparametric quantile regression models were used for the statistical analysis. In order to evaluate the robustness of the proposed reference charts regarding various distributional assumptions on the ultrasound measurements at hand, we compared the gestational age-specific reference curves we produced through the statistical methods used. Normal mean height based on parametric and nonparametric methods were defined for each week of gestation and the regression equation expressing the height of the cerebellar vermis as a function of gestational age was calculated. Finally the correlation between dimension/gestation was measured.

**Results:**

The mean height of the cerebellar vermis was 12.7mm (SD, 1.6mm; 95% confidence interval, 12.7–12.8mm). The regression equation expressing the height of the CV as a function of the gestational age was: height (mm) = -4.85+0.78 x gestational age. The correlation between dimension/gestation was expressed by the coefficient r = 0.87.

**Conclusion:**

This is the first prospective cross-sectional study on fetal cerebellar vermis biometry with such a large sample size reported in literature. It is a detailed statistical survey and contains new centile-based reference charts for fetal height of cerebellar vermis measurements.

## Introduction

New concepts regarding the anatomic development of the cerebellar vermis (CV) were recently introduced[1], changing the diagnostic approach of both CV and posterior fossa anomalies. These have improved the differentiation between normal and pathological conditions during fetal life [2][3]. Imaging of the fetal posterior fossa is an integral part of a routine anomaly scan[4]and its anomalies are associated in 75% of cases with other structural malformations, chromosomal and genetic diseases responsible for high mortality, cognitive, language and behavioral dysfunction among the children affected [5–8]. The posterior fossa is studied with axial planes which highlight the CV as an echoic structure between the two cerebellars hemispheres [9][10]. In these multiplanar images, the biometry of the cisterna magna, the closure of the 4^th^ ventricle and the transcerebellar diameter must be also evaluated, although the midsaggittal plane is the most important plane since it allows a better visualization of the CV [11].The prevalence of posterior fossa malformations diagnosed during the neonatal period is estimated to be 1:5000 live births [12] and posterior fossa malformations on imaging are now the most commonly diagnosed brain malformations in utero, though the actual incidence is unknown. The most frequent of these malformations, Blake’s pouch cyst (BPC), vermian hypoplasia and Dandy–Walker malformation(DWM), have a similar ultrasound appearance, but with a very different prognosis DWM is the most common disease with an incidence ranging from 1:25000 to 1:35000[13]. It is characterized by partial/complete agenesis of the CV, cystic dilation of the fourth ventricle, and an enlarged posterior fossa combined with a superior displacement of the cerebellar hemisphere [14].

The BPC is characterized by 1) normal anatomy and size of the vermis in a mid-sagittal section of fetal brain; 2) mild to moderate up-ward rotation of the vermis in amid-sagittal section of fetal brain; 3) normal size of the cisterna magna in both mid-sagittal and axial sections of fetal brain. Generally BPC has a normal neurodevelopmental outcome. Finally CV anomalies could be also present as an isolated finding with complete or partial vermian agenesis[15][16] causing extremely variable clinical manifestations during the neonatal period which range from mild to severe mental retardation and psychomotor delay [17–20].

Despite the well-defined ultrasonographic findings, there are still many misdiagnoses of both DWM,BPC and vermis isolated anomalies [11] and the most challenging differential diagnosis for BPC is the hypoplastic form of the CV, because it is based mainly on the size of the vermis. Hence, when a CV anomaly is suspected, the definition of CV size is crucial for the diagnosis of the partial/hypoplastic forms or BPC. Finally, the exclusion of associated structural malformations and Central Nervous System (CNS) defects remains the most important aspect of prenatal counseling and poses a challenge for all obstetricians.

With this in mind, we aim to provide reference charts of fetal CV height from a prospective cross-sectional study and to evaluate the robustness of the proposed reference charts when making various distributional assumptions.

## Materials and Methods

The institutional review board (Comitato Etico per la Ricerca Scientifica) approved the study and written informed consent was obtained from all patients (PRSV-09-0013–13/07/2009).

We carried out a prospective cross-sectional study between September 2009 and December 2014, at the ALTAMEDICA Fetal-Maternal Medical Centre, in Rome. We enrolled all the women who came to our institution who had requested a fetal biometric evaluation without making any selection of this population. The women involved in the study were between 20^+0^and35^+6^ weeks of gestation.

We excluded patients with hypertensive disorders, diabetes mellitus, multiple pregnancy, who had a fetus with an abnormal karyotype and/or congenital malformations, or no availability of first-trimester dating based on crown-rump length (CRL) [21]. Then, we excluded women with a history of congenital CNS abnormalities or those with fetuses suspected to be at risk for any CNS anomaly (potential maternal alcohol abuse, chromosomal abnormalities, single mutant genes, maternal diabetes mellitus, radiation exposure, congenital infections, and a parent or previous sibling with a neural defect). Finally, according to the cross-sectional rules, each fetus was included only once. If the CV of a fetus was measured more than once during pregnancy, only one measure was randomly chosen, while the remaining were not used to fit the statistical models. This was done to avoid any selection effect due to non-random choices or to considering all measurements for a single fetus, where fetuses with more than one measurement would be over-weighted.

All measurements were performed by eleven different gynecologists with at least 9 year of experience in fetal ultrasound and about 1000-scans/per-year. (MG, PC, FP, MM, LM, LD, VM, CB, RV, CG, CC). In all cases, General Electrics medical System Voluson 730 Pro or General Electrics medical System Voluson E8 with a 2D (4.5–16.5 MHz) trans-abdominal probe was used. When the visualization of the CV was difficult (i.e. high maternal body mass index or vertex presentation of the fetus), the exam was performed with a 2D (5–9 MHz) transvaginal probe. Each operator used both ultrasound systems during the study period.

**Fig 1:**
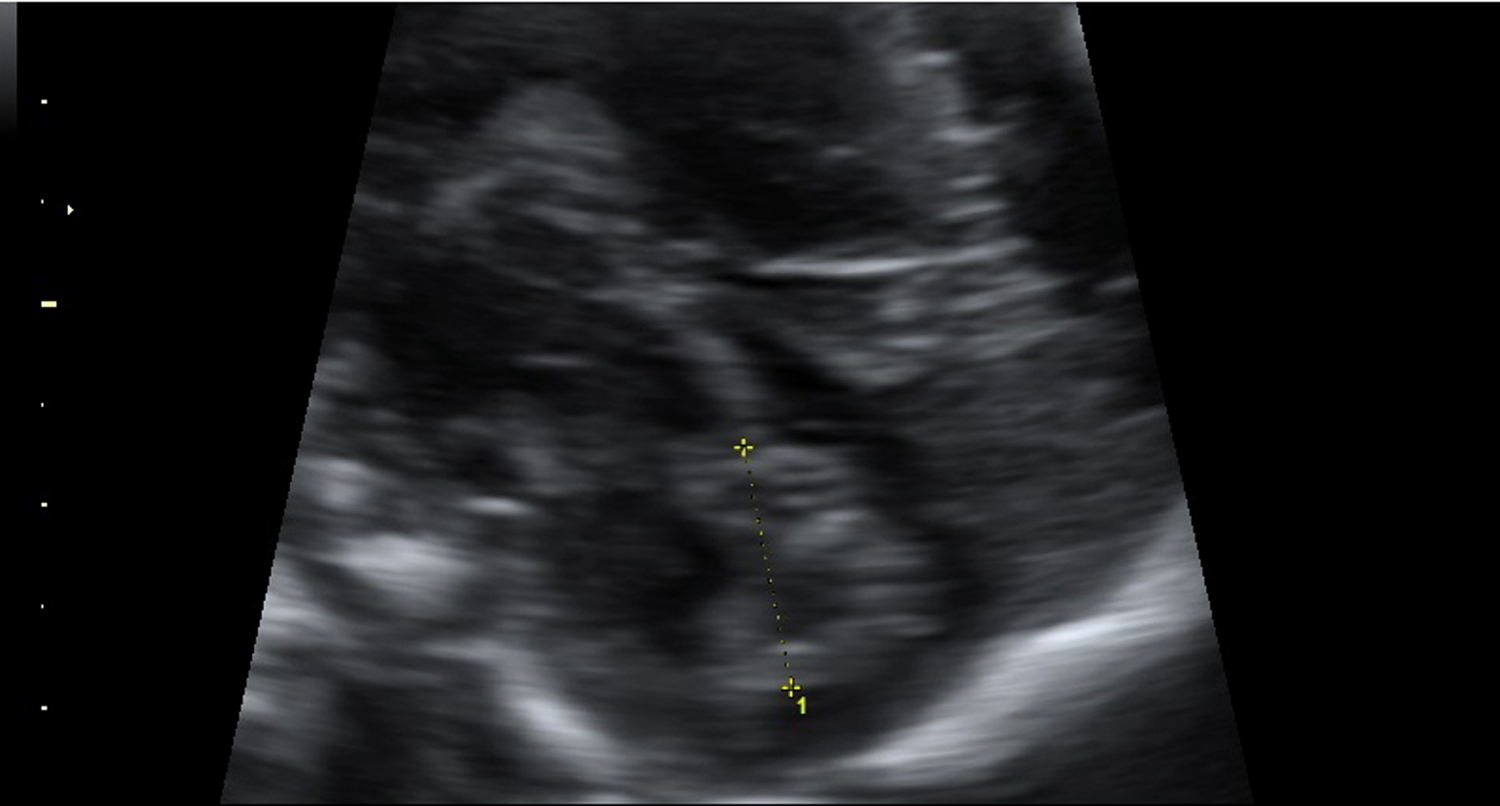
Measurement of the cerebellar vermis height.

The transducer was oriented basing on fetal head position. The head was usually viewed from a slightly posterior angle. This plane is obtained at a slightly lower level than that of transventricular plane and with a slight posterior tilting. An axial transcerebellar view was obtained at the level of the fourth cerebral ventricle. The beam was directed through the posterolateral (mastoid) fontanel to minimize shadowing. The depiction of the cerebellar vermis was based on the demonstration of serial axial planes with slight angulations between them, to demonstrate the portions of the cerebellar vermis. The cerebellar vermis was visualized in a mid-sagittal plane as an hyperechoic structure delimited anteriorly by the 4^th^ ventricle and posteriorly from the cisterna magna. In order to obtain a precise mid-sagittal plane, the corpus callosum should be clearly visualized anteriorly. The size of the cerebellar vermis was measured directly from a magnified view using 0.1 mm resolution. The vermis height was defined as the maximum distance between the most cranial portion of the culmen and the most caudal portion of the uvula.[22–24] (Fig 1).

The primary aim was to produce reference charts using the following statistical methods: a) Normal-based reference curves; b) Generalized Additive Models for Location, Scale and Shape (GAMLSS); c) Box-Cox t distribution and d) Nonparametric quantile regression. A further aim was to evaluate the robustness of the proposed reference charts when making various distributional assumptions on the ultrasound measurements at hand comparing the gestational age specific reference curve produced by the previously described statistical methods. Statistical analysis was performed using R 2.15.0 [25].In particular, the following packages were used: *quantreg*[26], *scam* [27], *gamlss*[28] and *ggplot2* [29].

### Normal-based reference curves

In spite of the recent trend towards nonparametric reference distributions, the Normal distribution or some simple transformation towards normality is still the most commonly used model. A key feature of such a model is the availability of standard deviation estimates and Z-scores, which represent the centile position corresponding to individual measurements transformed to the normal deviate scale, which cannot be calculated with quantile regression. If the model is correctly specified, the Z-scores follow, at least approximately, a standard Normal distribution independent of age. More specifically, Normal-based centile curves are given by μ_GA ±_ κσ_GA_ where μ_GA_ and σ_GA_ are, respectively, the gestational age (GA)-specific mean and standard deviation (SD) of (Y/ GA), while *k*is an appropriate percentage point of a standard Normal. The Z-score (or standardized residual) for (Y/ GA) is defined as (Y- μ_GA_)/σGA. Suitable regression functions may be used to model μ_GA_ and σ_GA_ with parameters estimated by, for example, maximum likelihood. As suggested by Royston and Wright [30], in this paper we have considered t cubic polynomials to model μ_GA_ and σ_GA_, adopting the Akaike Information Criterion (AIC) to select the more appropriate model. More in detail, to estimate σ_GA_, we have regressed the so called “scaled absolute residuals”–that is, the absolute residuals multiplied by $\sqrt{\pi /2}$ – on GA. Standard diagnostic plots and tools were also considered.

### GAMLSS and Box-Cox t distribution

Usually, data may require a suitable transformation to achieve approximate normality. Since biometric measurements tend to follow a (positively) skewed normal distribution at a given GA, the data are usually log-transformed. More in general, the LMS method [31] uses the power transformation family of Box and Cox to allow the skewness of the measurement distribution, as well as the median and variability, to vary with age. Given the marked leptokurtosis shown by the observed data, we adopt an extension of the original method devised by Rigby and Stasinopoulos [32] which is based on the so-called Box–Cox t (BCT) distribution. This distribution is defined by a power transformation of the original variable leading to a shifted and scaled t distribution with degrees of freedom parameter τ. This produces a model with four parameters usually denoted by BCT(μ,σ,ν,τ). The parameters μ, σ, ν and τ may be related to location (median), scale (centile-based coefficient of variation), skewness (power transformation to symmetry) and kurtosis (degrees of freedom), respectively. The generalized additive model for location, scale and shape (GAMLSS) allows each of the parameters to be modeled as linear or non-linear, parametric or smooth nonparametric functions of explanatory variables such as gestational age. As mentioned above, we consider cubic polynomials to model μ, σ, ν and τ as a function of GA, adopting the (generalized) AIC criterion for model selection. Once a BCT-GAMLSS is fitted, we use the estimates (μ,σ,ν,τ) to obtain the centile curve y_q_ at any level q Є (0,1) for the variable measured on the original scale, by adopting an explicit back-transformation; that is, by simply substituting the estimated values into

$$y_{q}=\{\begin{array}{c} \hat{\mu }{\left[1+\hat{\sigma }\hat{\nu }t_{\hat{\tau },q}\right]}^{1/\hat{\nu }},\;\text{if}\;\hat{\nu }\neq 0;\\ \hat{\mu }\;\text{exp}\left\{\hat{\sigma }t_{\hat{\tau },q}\right\},\;\text{if}\;\hat{\nu }=0. \end{array}$$

Here $t_{\hat{\tau },q}$ is the100*q*centile of a standard t distribution with $\hat{\tau }$ degrees of freedom, and we have suppressed the dependence of model parameters on GA. From a diagnostic point of view, we first considered the worm plot [33], a tool to visualize how well a statistical model fits the data. It consists of a number of detrended normal Q-Q plots of the residuals, split according to pre-specified classes of gestational age. A model that fits the data well is characterized by “flat worms”. The worm plot makes it possible to detect inadequacies in model fit within each specific class of GA. The fit within GA classes can also be further investigated by calculating *Q*statistics to test for normality of the residuals within each group. More specifically, we assume *K* to be the number of GA classes and let {*e*_*k*,*i*_, *i* = 1,…,*n*_*k*_} be the residuals in group *k*, for *k* = 1,…,*K*. Statistics $\left(Z_{k}^{\mu },Z_{k}^{\sigma },Z_{k}^{\nu },Z_{k}^{\tau }\right)$ are calculated from the residuals in group κto test whether they have mean 0, variance 1, skewness 0 and kurtosis 3. Finally, the *Q* statistics (Q^μ^, Q^σ^, Q^ν^, Q^Г^) of Royston and Wright [34] are calculated as the within group sum of squares of the corresponding *Z*’s. Significant *Q*’s statistics indicate potential lack of fit for models describing parameters μ, σ, ν and τ respectively.

### Nonparametric quantile regression

Quantile regression [35] is a general non-parametric approach which can be used to estimate age-specific reference intervals. The QR model is defined to minimize the following objective function

$$\text{L}\left(\beta \right)=\Sigma_{\text{j}}\rho_{q}({\text{y}}_{\text{j}\;-}\;\beta ),$$

over the scalar β, where ρ_*q(u) = (q-I(u<0))*_ is the so called “check function” and I(A) denotes the indicator function of the set *A*. The optimal solution β corresponds to the *q*^th^ sample quantile of the observed data {*y*_1_,…,*y*_*n*_};that is $\beta =F_{y}^{-1}(q)$ with *F*_*y*_(⋅) being the cumulative distribution function of y. This basic framework has been extended in many directions. For what concerns the present study, the most relevant, consists in replacing β with a smooth, possibly shape-constrained, function *g*(⋅) of some explanatory variables, adding a suitable smoothing penalty term to the objective function *L*(⋅). In the present work, in order to reduce the variability of the estimated reference curves and to avoid any artifact due to the scarcity of data at earlier or later gestational ages, we first fitted a non-decreasing smooth quantile function of the gestational age as implemented in the *quantreg* package. Due to the total variation smoothing penalty adopted, this procedure results in piecewise linear curves with knots at the observed data. To get a smoother–locally quadratic, for example–fit, we also adapted the iterative procedure proposed by Hee-Seok et al [36]to our shape-constrained framework. Diagnostics were again based on a recent adaptation of the worm plot to the QR setup [37], whereas uniform and point wise confidence bands have also been provided to better understand how variable (and consequently reliable) the obtained estimates are.

## Results and Discussion

Among 25203 fetal measurements during the study period, 12167(48%)measurements of the fetal CV height were available. We excluded 280 (2.3%)spontaneous abortions, 330(2.7%)major fetal abnormalities, 170 (1.4%)twin pregnancies, 198(1.6%)women with diabetes, and 360(2.9%)women with hypertensive disorders. Then we excluded 224 (1,8%)repeated measurements, and a total of 10605 (87%) fetuses were identified.

Five thousand one hundred and ninety-six fetuses (49%) were in a vertex presentation, and 5409 (51%) were in a breech presentation. Among fetuses in the vertex presentations, 1641 (31.6%) were examined vaginally, and 3555 (68.4%) abdominally. In five cases (3.5%) a combined approach was used. In both the vertex and breech group, there were 100% cases with successful views of the vermis. Baseline characteristics are shown in Table 1.

**Table 1:** Demographic and baseline characteristics.

VARIABLES	GROUP
Age (years)	33.8 ± 4.4
BMI (Kg/m^2^)	22.1 (22.4–24.6)
Ethnic origin	
caucasian	10541 (99.4%)
asiatic	42 (0.4%)
African	22 (0.2%)
Smoker	498 (4.7%)
Primipara	4772 (45%)
Gestational age (weeks)	22.4 ±1.87

Data are median (IQR), number (%), or mean ±SD

The mean CV height was 12.7mm (SD, 1.6mm; 95% CI, 12.7–12.8mm). The distribution of mean heights and standard deviations by gestational age (GA) is shown in Table 2.

**Table 2:** Fetal Cerebellar Vermis Height (mm) according to gestational age.

GA	n	M	SD	95% CI
20^+0^–20^+6^	636	11.27	0.58	10.69–11.85
21^+0^–21^+6^	4549	11.96	0.67	11.29–12.63
22^+0^–22^+6^	4160	12.71	0.76	11.95–13.47
23^+0^–23^+6^	692	13.50	0.85	12.65–14.35
24^+0^–24^+6^	89	14.32	0.94	13.38–15.26
25^+0^–25^+6^	66	15.16	1.03	14.13–16.19
26^+0^–26^+6^	56	16.01	1.12	14.89–17.13
27^+0^–27^+6^	52	16.85	1.21	15.64–18.06
28^+0^–28^+6^	36	17.67	1.30	16.37–18.97
29^+0^–29^+6^	47	18.47	1.39	17.08–19.86
30^+0^–30^+6^	51	19.22	1.48	17.74–20.70
31^+0^–31^+6^	77	19.91	1.57	18.34–21.48
32^+0^–32^+6^	53	20.54	1.66	18.88–22.20
33^+0^–33^+6^	26	21.09	1.75	19.34–22.84
34^+0^–34^+6^	6	21.54	1.84	19.70–23.38
35^+0^–35^+6^	9	21.90	1.93	19.97–23.82

GA, Week^+days^; n, number of fetuses; M, mean in millimeters; SD, standard deviation; CI, confidence interval.

The intra-observer variability showed a low average coefficient of variation, ranging from 5.6% to 6%; whereas, the inter-observer variability among the sonographers was very low. In particular, between 20 and 24 weeks of gestation, when most of the measurements were taken, the largest per week observed mean difference went from 0.4 to 0.8mm, corresponding to a modest relative variation among sonographers of 3.6% up to 5.9% respectively.

We now provide the results obtained by the three approaches previously described.

### Normal-Based Reference Curves

To start with, we looked at the usual normal-based reference curves. The linear model selected by the Akaike Information Criterion consists of a cubic polynomial with the following regression equations:

$$\begin{array}{l} \mu_{\text{GA}}=42.891\;-\;4.817\;\times \;\text{GA}\;+\;0.216\;\times \;{\text{GA}}^{2}\;-\;0.003\;\times \;{\text{GA}}^{3};\\ \sigma_{\text{GA}}=-\;1.219\;+\;0.088\;\times \;\text{GA}. \end{array}$$

**Fig 2:**
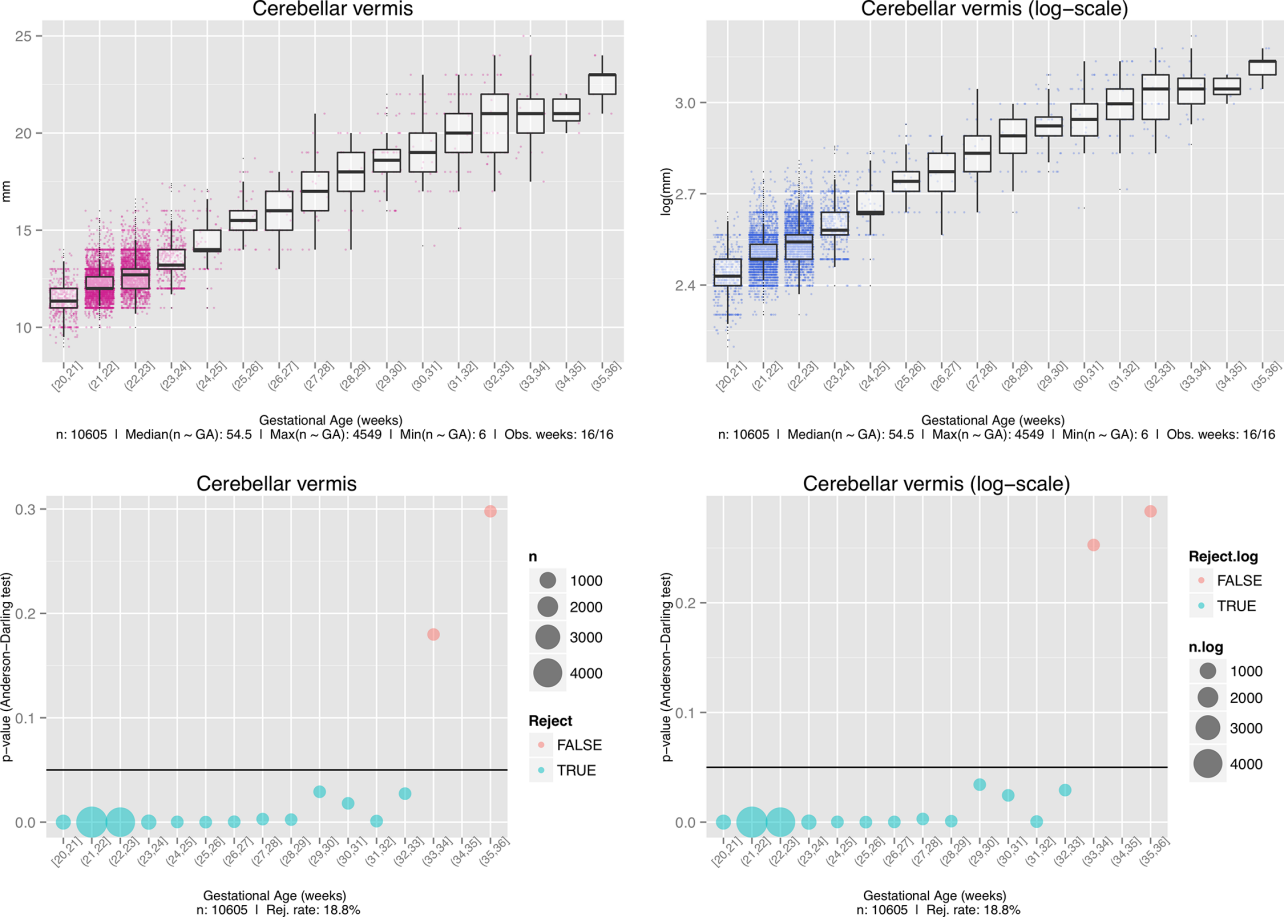
Anderson-Darling tests and the associated diagnostic plots. *Top left*, Box plot of the original measurements. *Bottom left*, Raw p-values associated with the Anderson-Darling test of normality for the original measurements. *Top right*, Box plot of the log-transformed data. *Bottom right*, Raw p-values associated with the Anderson-Darling test of normality for the log-transformed data. The sizes of the circles in the bubble plot are proportional to the numbers of samples (n) available during that week.

All of the covariate effects were strongly significant, although this result has to be handled with care in light of the Anderson-Darling tests and the associated diagnostic plots shown in Fig 2. In fact, it is clear that, mainly due to the strong leptokurtosis shown by the data, and even after the usual log transformation, normality cannot be assumed over the whole gestational period.

**Fig 3:**
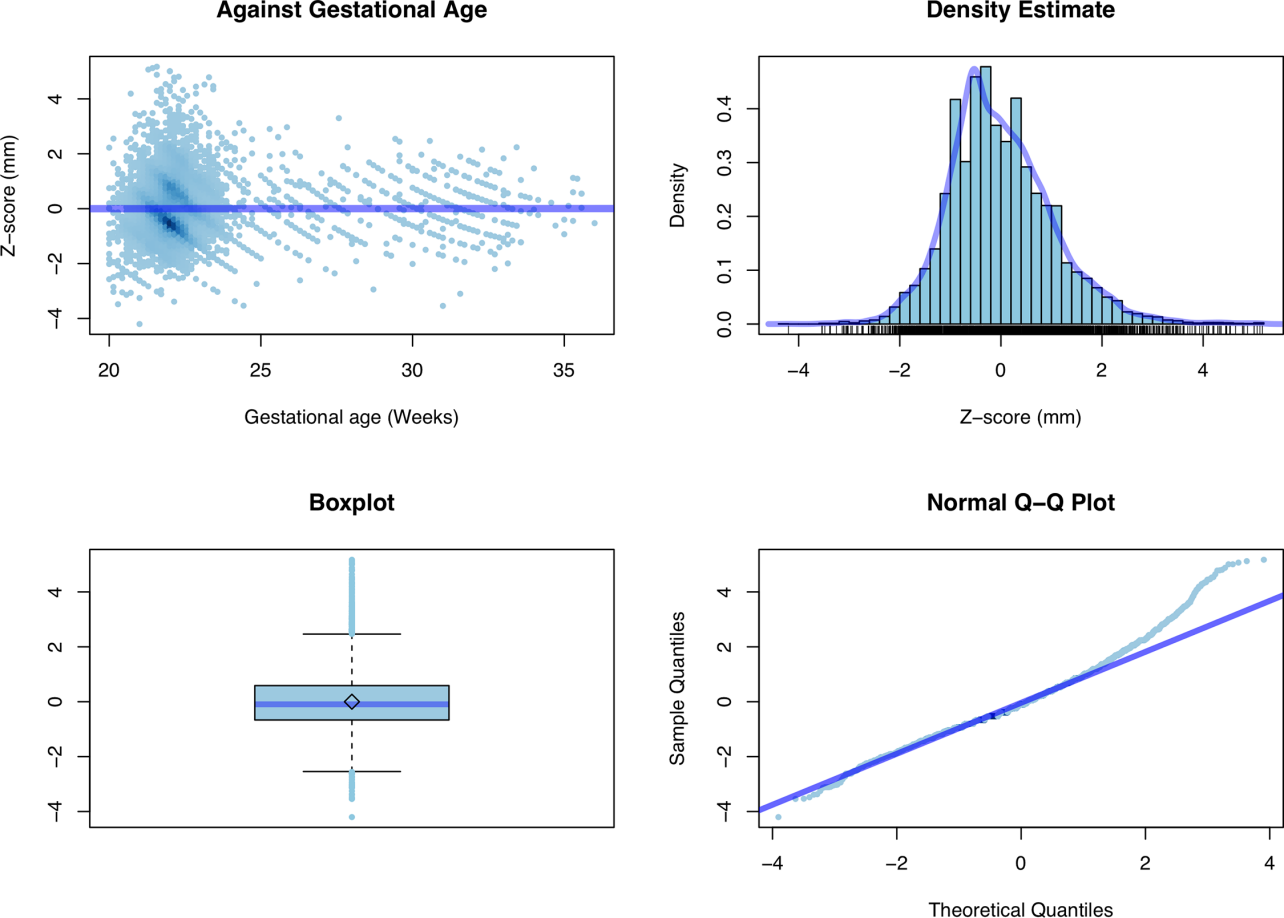
Normal-based mean curve. 95^th^ and 5^th^ centile curves across gestational age.

**Fig 4:**
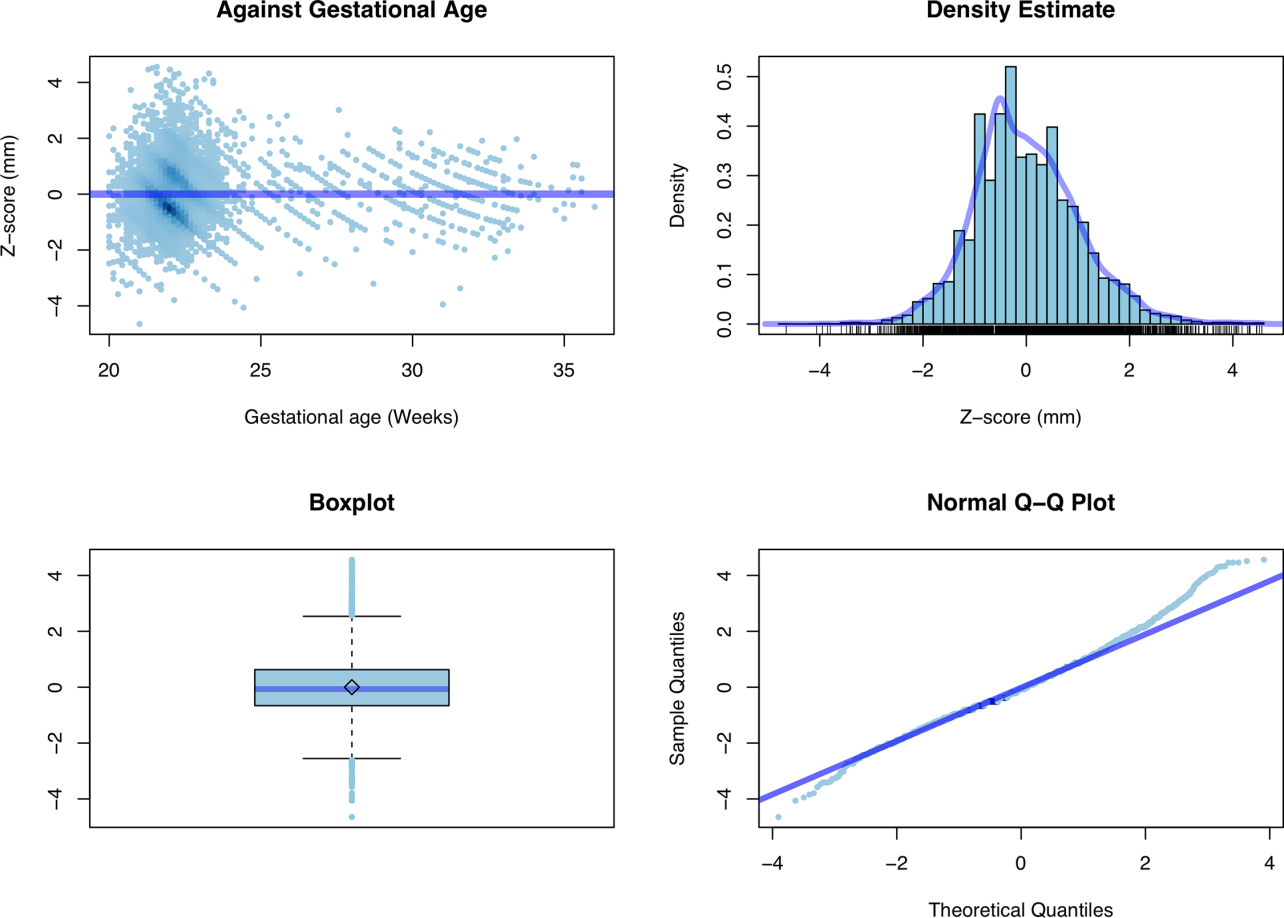
Z score. Diagnostic plots for the original measurements.

**Fig 5:**
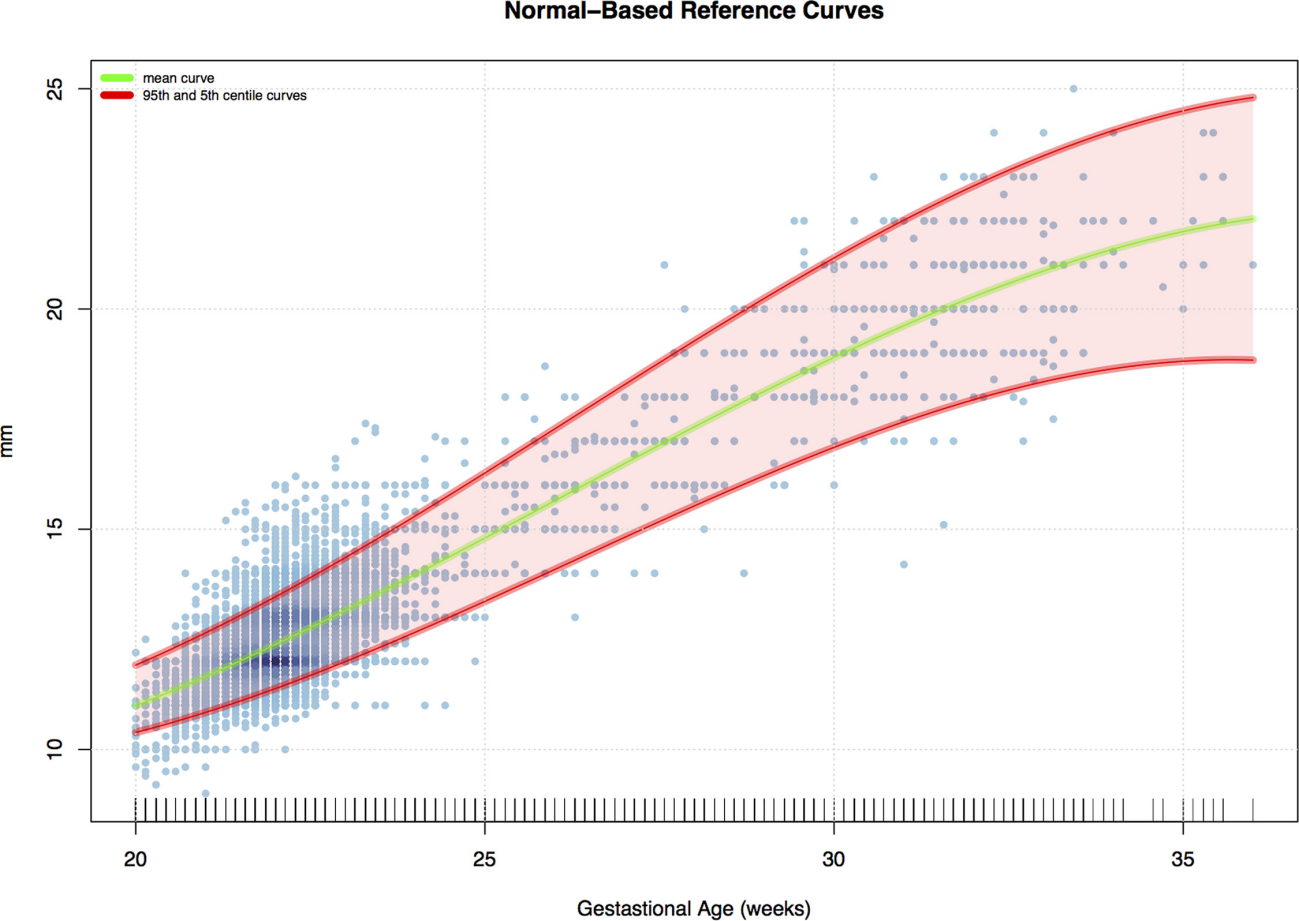
Z score. Diagnostic plots for the log-transformed data.

Fig 3 shows the estimated mean curve together with 95^th^ and 5^th^ centile curves, whereas Fig 4 and Fig 5 contain some standard diagnostic plots for the Z scores associated with the original data and their log-transformed versions. As mentioned above, the empirical distribution of the original data is characterized by heavier tails than the normal distribution and a marked positive skewness which is only mildly mitigated when working on the log scale.

In order to get a better fit, in the next section, we discuss the results of the (polynomial) GAMLSS model based on the BCT distribution.

### GAMLSS and BCT Distribution

To effectively capture the evident kurtosis shown by the data, we considered a GAMLSS-BCT model with, cubic polynomials to model μ, σ, ν and τ as functions of GA. The model selected by the (generalized) Akaike Information Criterion was then specified by the following set of regression equations:

$$\begin{array}{l} \mu_{\text{GA}}=12.67\;+\;143.70\;\times \;\text{GA}\;+\;1.96\;\times \;{\text{GA}}^{2}\;-\;5.25\;\times \;{\text{GA}}^{3};\\ \sigma_{\text{GA}}=-\;2.67\;+\;10.61\;\times \;\text{GA}\;-\;7.13\;\times \;{\text{GA}}^{2};\\ \nu_{\text{GA}}=-\;2.28\;+\;79.82\;\times \;\text{GA}\;-\;13.79\;\times \;{\text{GA}}^{2};\\ \tau_{\text{GA}}=3.07\;+\;91.90\;\times \;\text{GA}\;-\;15.96\;\times \;{\text{GA}}^{2}+\;49.48\;\times \;{\text{GA}}^{3}. \end{array}$$

**Fig 6:**
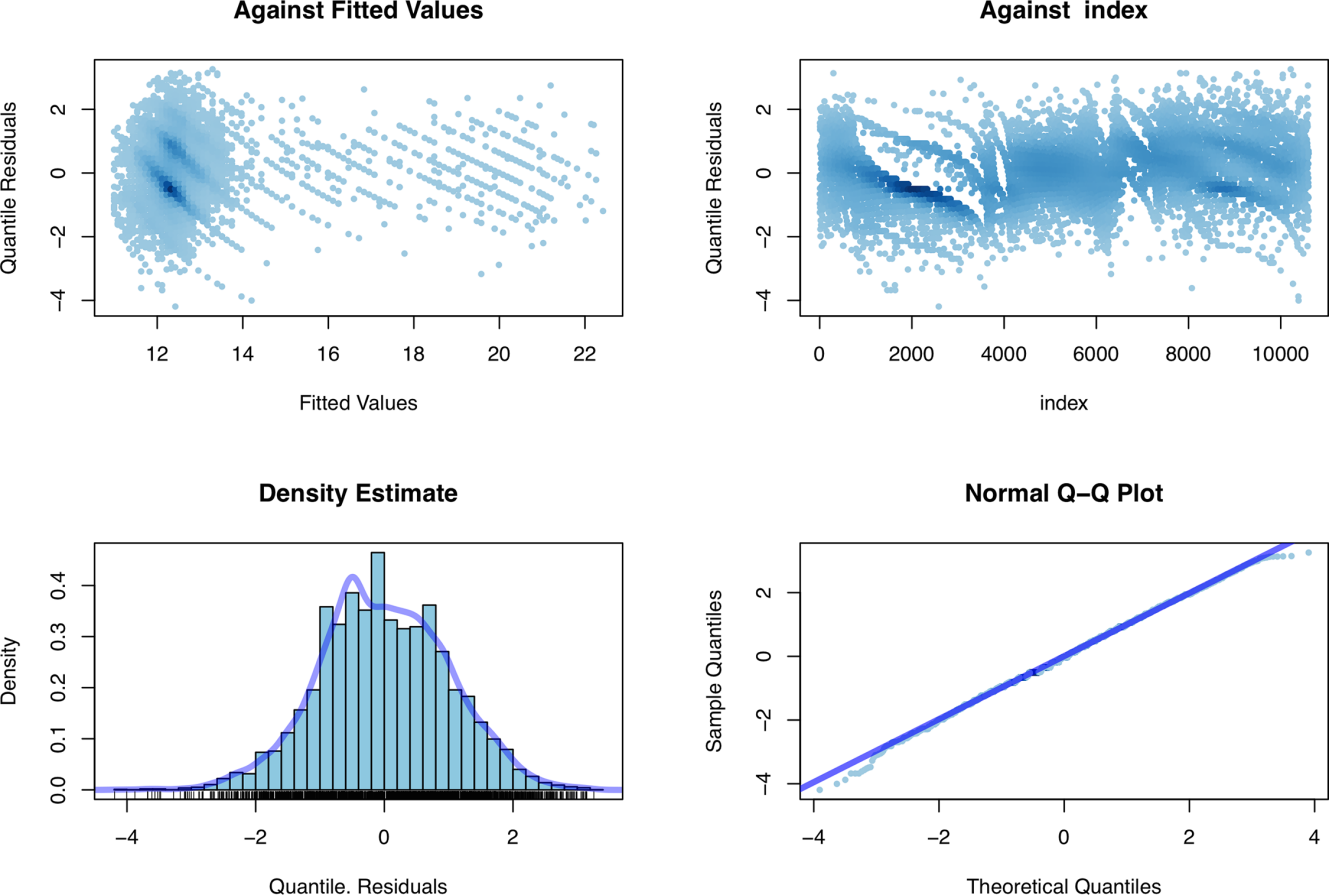
GAMLSS diagnostic plots.

Fig 6 displays the (normalized) quantile residuals corresponding to the chosen model. The top panels plot the residuals against the fitted values for μ and versus GA, respectively, whereas the bottom panels provide a kernel density estimate and a normal Q-Q plot in order to visualize departures from normality. The residuals appear to be essentially random (mean, –0.005; variance, 0.999; coefficient of skewness, –0.009; coefficient of kurtosis, 3.006), with only few outliers. In conclusion, we can say the BCT model provides a very good fit for the data at hand, and we can confidently use the fitted values (μ,σ,ν,τ) to obtain the centile curves at different levels q Є (0,1) of interest.

### Nonparametric Quantile Regression

**Fig 7:**
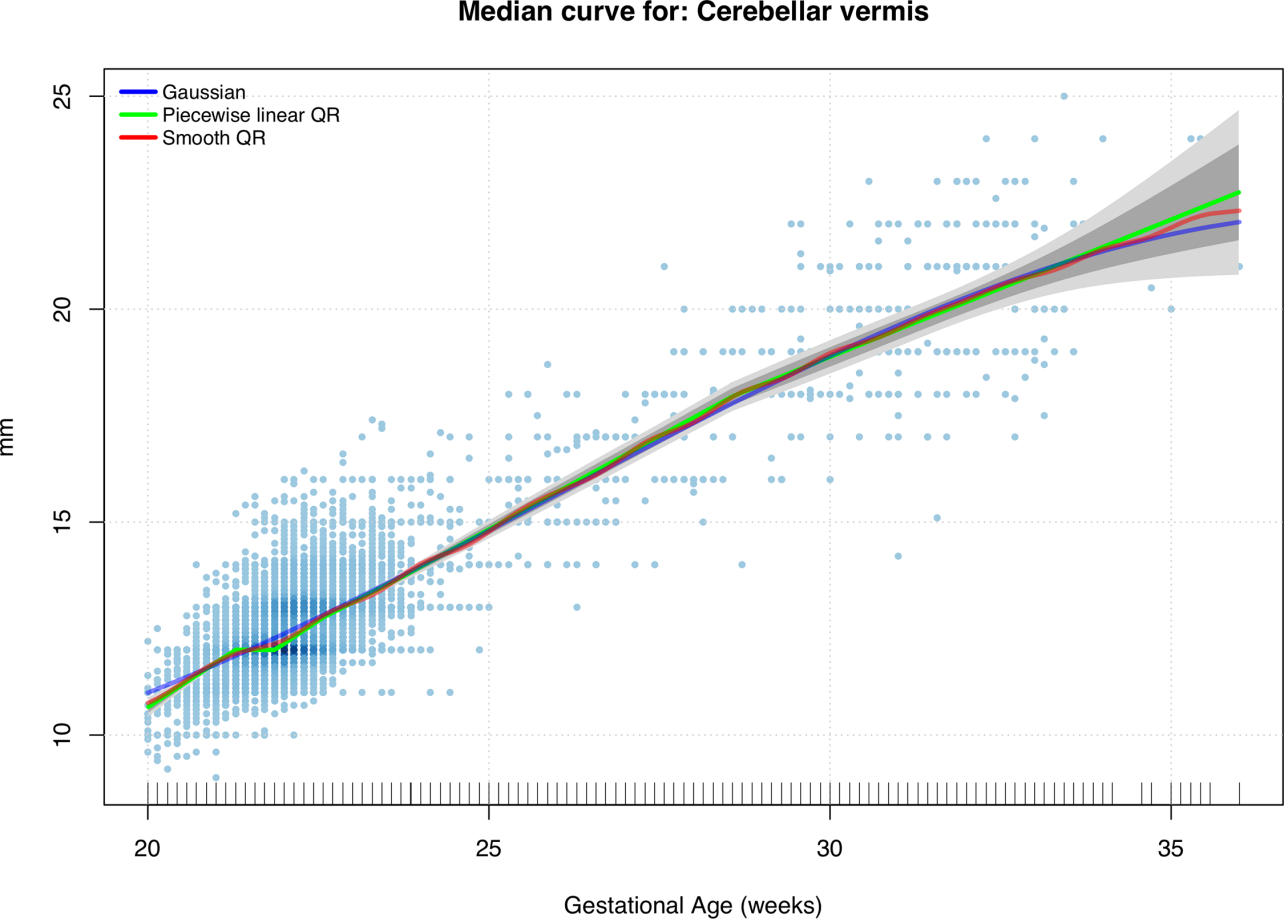
Shape-constrained quantile regression (QR). Estimates for the conditional median and approximate confidence bands (point-wise bands in dark gray, uniform bands in light gray) compared with the standard normal-based fit.

**Fig 8:**
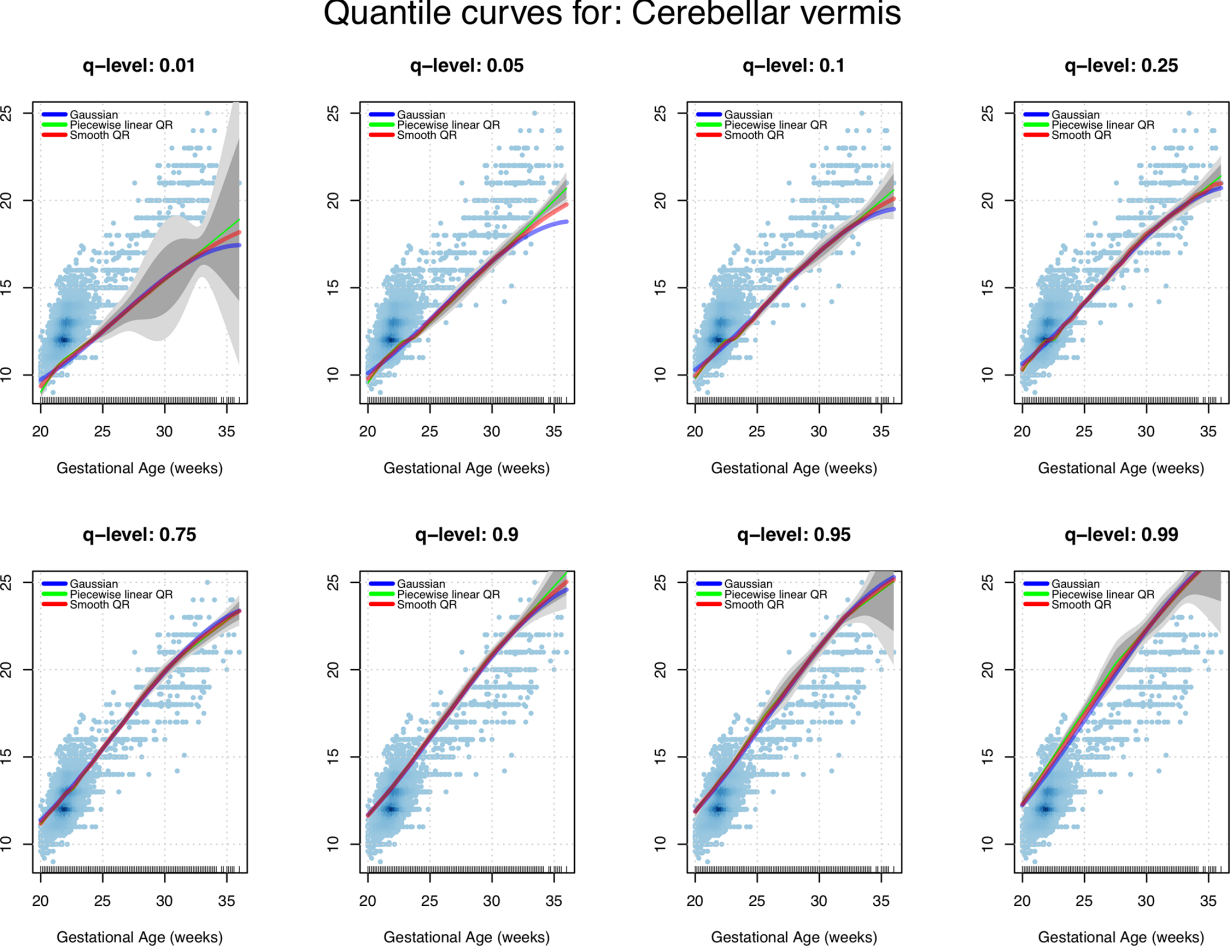
Shape-constrained quantile regression (QR). Estimates for a range of levels of interest and approximate confidence bands (point-wise bands in dark gray, uniform bands in light gray) compared with the standard normal-based fit.

We now consider a shape-constrained (nondecreasing) spline-based quantile regression model. The introduction of specific shape information (e.g. convexity and monotonicity) to drive the fit and reduce its overall variability, was essentially needed to avoid unrealistic behaviors of the more extreme centile curves induced by the scarcity of data at later GAs. As mentioned above, we examined a piecewise linear fit provided by the *quantreg* package and a smoother (quadratic) alternative. Figs 7 and 8 show the resulting centile curves and compare them with the standard normal-based solution. The (generalized) worm plots were associated with the fitted linear and the quadratic quantile regression models. Regarding the GAMLSS solution, even in this case, there was no strong evidence of a lack of fit.

### Comparative Tables

In Tables 3–6, we first summarize the estimated reference curves for the normal-based method, the piecewise linear quantile regression, the “smooth” quantile regression, and the BCT GAMLSS centile. Then, in Table 7, we provide the median absolute deviation for the predictions obtained at different GAs and quantiles by these four techniques in order to quantify their overall agreement.

In this study, we established new reference charts for fetal CV height deriving from the largest prenatal prospective cross-sectional study ever performed. In this paper, we provide a comparison of different statistical methods to evaluate the robustness of the proposed reference charts to be used when making various distributional assumptions on the ultrasound measurements.

**Table 3:** Reference Table for Fetal Cerebellar Vermis Height (mm): Normal-Based Model.

t	Quantile									
GA	n	0.50	0.01	0.05	0.10	0.25	0.75	0.90	0.95	0.99
20^+0^–20^+6^	636	11.27	9.92	10.32	10.53	10.88	11.66	12.02	12.23	12.62
21^+0^–21^+6^	4549	11.96	10.40	10.86	11.10	11.51	12.42	12.82	13.07	13.52
22^+0^–22^+6^	4160	12.71	10.94	11.46	11.74	12.20	13.22	13.69	13.96	14.48
23^+0^–23^+6^	692	13.50	11.52	12.10	12.41	12.93	14.08	14.59	14.90	15.48
24^+0^–24^+6^	89	14.32	12.14	12.78	13.12	13.69	14.96	15.53	15.87	16.51
25^+0^–25^+6^	66	15.16	12.77	13.47	13.84	14.47	15.86	16.48	16.86	17.56
26^+0^–26^+6^	56	16.01	13.40	14.17	14.58	15.26	16.77	17.45	17.85	18.62
27^+0^–27^+6^	52	16.85	14.04	14.86	15.30	16.04	17.67	18.40	18.84	19.67
28^+0^–28^+6^	36	17.67	14.65	15.54	16.01	16.80	18.55	19.34	19.81	20.70
29^+0^–29^+6^	47	18.47	15.23	16.18	16.69	17.53	19.40	20.25	20.75	21.70
30^+0^–30^+6^	51	19.22	15.78	16.78	17.32	18.22	20.21	21.11	21.65	22.66
31^+0^–31^+6^	77	19.91	16.26	17.33	17.90	18.85	20.97	21.92	22.49	23.56
32^+0^–32^+6^	53	20.54	16.68	17.81	18.41	19.42	21.66	22.67	23.27	24.40
33^+0^–33^+6^	26	21.09	17.02	18.21	18.85	19.91	22.27	23.33	23.96	25.16
34^+0^–34^+6^	6	21.54	17.27	18.52	19.19	20.30	22.78	23.90	24.57	25.82
35^+0^–35^+6^	9	21.90	17.41	18.72	19.42	20.59	23.20	24.37	25.07	26.38

GA, GA, Week^+days^; n, number of fetuses;

**Table 4:** Reference Table for Fetal Cerebellar Vermis Height (mm): Piecewise Linear Quantile Regression Model.

	Quantile									
GA	n	0.50	0.01	0.05	0.10	0.25	0.75	0.90	0.95	0.99
20^+0^–20^+6^	636	11.29	9.50	10.00	10.23	10.66	11.52	12.00	12.27	12.83
21^+0^–21^+6^	4549	12.00	10.61	11.00	11.17	11.64	12.36	12.83	13.20	13.89
22^+0^–22^+6^	4160	12.65	11.19	11.70	12.00	12.00	13.07	13.73	14.11	14.96
23^+0^–23^+6^	692	13.40	11.71	12.05	12.18	12.88	14.00	14.69	15.10	16.02
24^+0^–24^+6^	89	14.30	12.22	12.60	13.00	13.68	14.93	15.64	16.16	17.09
25^+0^–25^+6^	66	15.20	12.77	13.30	13.82	14.48	15.87	16.59	17.22	18.15
26^+0^–26^+6^	56	16.10	13.35	14.00	14.64	15.28	16.80	17.55	18.12	19.21
27^+0^–27^+6^	52	17.00	13.93	14.70	15.46	16.09	17.74	18.50	19.00	20.28
28^+0^–28^+6^	36	17.90	14.51	15.40	16.08	16.89	18.67	19.46	19.88	21.10
29^+0^–29^+6^	47	18.55	15.10	16.10	16.68	17.69	19.48	20.41	20.75	21.89
30^+0^–30^+6^	51	19.15	15.68	16.80	17.28	18.28	20.19	21.23	21.63	22.67
31^+0^–31^+6^	77	19.76	16.26	17.50	17.87	18.84	20.84	22.00	22.50	23.45
32^+0^–32^+6^	53	20.36	16.84	18.20	18.47	19.40	21.40	22.78	23.17	24.22
33^+0^–33^+6^	26	20.97	17.42	18.90	19.06	19.96	21.96	23.56	23.70	25.00
34^+0^–34^+6^	6	21.57	18.00	19.59	19.66	20.52	22.52	24.33	24.23	25.78
35^+0^–35^+6^	9	22.18	18.58	20.29	20.26	21.08	23.08	25.11	24.75	26.55

GA, Week^+days^; n, number of fetuses

**Table 5:** Reference Table for Fetal Cerebellar Vermis Height (mm): GAMLSS Centile Estimates.

	Quantile									
GA	n	0.50	0.01	0.05	0.10	0.25	0.75	0.90	0.95	0.99
20^+0^–20^+6^	636	11.25	9.12	10.14	10.49	10.89	11.65	12.27	12.94	16.08
21^+0^–21^+6^	4549	11.92	10.63	11.01	11.21	11.54	12.35	12.83	13.19	14.13
22^+0^–22^+6^	4160	12.64	11.20	11.59	11.81	12.18	13.15	13.69	14.06	14.90
23^+0^–23^+6^	692	13.40	11.71	12.16	12.41	12.86	14.01	14.64	15.06	15.96
24^+0^–24^+6^	89	14.21	12.20	12.73	13.03	13.57	14.92	15.63	16.10	17.08
25^+0^–25^+6^	66	15.03	12.68	13.32	13.67	14.29	15.84	16.64	17.15	18.21
26^+0^–26^+6^	56	15.87	13.16	13.91	14.32	15.04	16.77	17.64	18.19	19.30
27^+0^–27^+6^	52	16.72	13.64	14.51	14.98	15.79	17.69	18.61	19.19	20.33
28^+0^–28^+6^	36	17.55	14.15	15.13	15.66	16.55	18.58	19.54	20.13	21.28
29^+0^–29^+6^	47	18.37	14.70	15.78	16.35	17.31	19.43	20.41	21.00	22.13
30^+0^–30^+6^	51	19.15	15.32	16.47	17.07	18.06	20.23	21.20	21.77	22.86
31^+0^–31^+6^	77	19.89	16.00	17.18	17.79	18.80	20.96	21.90	22.46	23.50
32^+0^–32^+6^	53	20.58	16.73	17.91	18.52	19.51	21.61	22.52	23.05	24.03
33^+0^–33^+6^	26	21.20	17.51	18.65	19.23	20.18	22.18	23.03	23.53	24.45
34^+0^–34^+6^	6	21.75	18.32	19.38	19.92	20.80	22.65	23.44	23.90	24.75
35^+0^–35^+6^	9	22.20	19.12	20.07	20.56	21.35	23.02	23.74	24.15	24.92

GA, Week^+days^; n, number of fetuses

**Table 6:** Reference Table for Fetal Cerebellar Vermis Height (mm): Smooth Quantile Regression Model.

	Quantile									
GA	n	0.50	0.01	0.05	0.10	0.25	0.75	0.90	0.95	0.99
20^+0^–20^+6^	636	11.14	9.70	10.12	10.34	10.77	11.59	12.00	12.24	12.73
21^+0^–21^+6^	4549	11.97	10.50	10.94	11.13	11.61	12.37	12.83	13.13	13.71
22^+0^–22^+6^	4160	12.66	11.07	11.60	11.86	12.08	13.14	13.71	14.04	14.72
23^+0^–23^+6^	692	13.41	11.62	12.07	12.26	12.92	14.06	14.63	15.00	15.75
24^+0^–24^+6^	89	14.29	12.16	12.69	13.02	13.68	14.93	15.61	16.03	16.80
25^+0^–25^+6^	66	15.25	12.78	13.39	13.88	14.47	15.90	16.55	17.06	17.86
26^+0^–26^+6^	56	15.99	13.38	14.08	14.60	15.24	16.75	17.47	17.98	18.92
27^+0^–27^+6^	52	16.99	13.99	14.79	15.41	16.12	17.73	18.44	18.91	19.96
28^+0^–28^+6^	36	17.79	14.57	15.47	16.05	16.87	18.62	19.42	19.83	20.91
29^+0^–29^+6^	47	18.46	15.17	16.12	16.67	17.60	19.43	20.35	20.78	21.81
30^+0^–30^+6^	51	19.19	15.73	16.81	17.32	18.28	20.22	21.20	21.65	22.67
31^+0^–31^+6^	77	19.87	16.25	17.39	17.88	18.85	20.89	21.97	22.48	23.50
32^+0^–32^+6^	53	20.51	16.76	18.00	18.45	19.37	21.55	22.73	23.22	24.30
33^+0^–33^+6^	26	21.00	17.22	18.57	18.93	19.94	22.06	23.43	23.84	25.07
34^+0^–34^+6^	6	21.58	17.64	19.09	19.47	20.41	22.58	24.09	24.37	25.79
35^+0^–35^+6^	9	22.19	18.00	19.53	19.89	20.90	23.10	24.69	24.89	26.48

GA, Week^+days^; n, number of fetuses

**Table 7:** Median Absolute Deviation (mm) for the predictions obtained at different GAs and quantiles by the four methods considered.

	Quantile									
GA	0.50	0.01	0.05	0.10	0.25	0.75	0.90	0.95	0.99	Average
20^+0^–20^+6^	0.10	0.31	0.10	0.14	0.09	0.05	0.01	0.03	0.16	0.11
21^+0^–21^+6^	0.03	0.10	0.05	0.05	0.07	0.01	0.00	0.05	0.27	0.07
22^+0^–22^+6^	0.04	0.10	0.08	0.09	0.09	0.06	0.01	0.05	0.18	0.08
23^+0^–23^+6^	0.05	0.07	0.04	0.11	0.04	0.04	0.04	0.07	0.20	0.07
24^+0^–24^+6^	0.03	0.04	0.07	0.02	0.01	0.01	0.02	0.10	0.21	0.06
25^+0^–25^+6^	0.07	0.01	0.07	0.04	0.01	0.02	0.07	0.12	0.26	0.07
26^+0^–26^+6^	0.07	0.04	0.13	0.04	0.03	0.01	0.07	0.16	0.28	0.09
27^+0^–27^+6^	0.10	0.08	0.12	0.12	0.06	0.04	0.07	0.12	0.27	0.11
28^+0^–28^+6^	0.12	0.10	0.10	0.05	0.07	0.05	0.09	0.05	0.27	0.10
29^+0^–29^+6^	0.04	0.10	0.06	0.01	0.12	0.02	0.04	0.02	0.14	0.06
30^+0^–30^+6^	0.03	0.07	0.02	0.03	0.04	0.01	0.02	0.01	0.01	0.03
31^+0^–31^+6^	0.03	0.01	0.13	0.02	0.01	0.06	0.05	0.01	0.04	0.04
32^+0^–32^+6^	0.05	0.06	0.14	0.04	0.04	0.08	0.08	0.07	0.13	0.08
33^+0^–33^+6^	0.07	0.21	0.24	0.16	0.04	0.16	0.17	0.19	0.12	0.15
34^+0^–34^+6^	0.13	0.50	0.37	0.33	0.16	0.10	0.32	0.25	0.03	0.24
35^+0^–35^+6^	0.14	0.83	0.56	0.50	0.33	0.06	0.55	0.24	0.13	0.37
**Average**	0.07	0.16	0.14	0.11	0.08	0.05	0.10	0.10	0.17	

GA, Week^+days^

Although over the years a variety of strategies have been published concerning the construction of reference charts [38], unreliable methods have still been used for fetal measurements of all biometric parameters [30] and to the best of our knowledge, few studies have reported reference ranges for the fetal CV [22–24][39]. Infact, the choice of reference charts and equations from a sample which is as similar as possible to the screened population may have a significant impact on the quality of obstetrical practice and patient counseling to assess observed fetal biometric parameters [40].Therefore, the analysis was based on different techniques, which produced substantially similar results (Table 7). From Figs 7 and 8, Tables 3 and 4 and Table 6, in fact, we can see the good agreement between the (strongly parametric) normal-based curves and the more data-driven quantile regression solution. This is particularly evident when we look between20 and 24 weeks of gestation when most of the measurements were performed. Considering the balance between easier techniques and more flexible statistical methods, this suggests that, in spite of its evident lack-of-fit, reference tables produced by the simple normal-based technique are definitively reliable when adopted during the standard sonographic screening weeks. On the other hand, when the focus is on extreme centiles associated to later gestational weeks when data are scarce, it is definitively more advisable to rely on reference intervals produced by intrinsically robust methods such as the shape-constrained nonparametric quantile regression model adopted in this work.

Naturally, one weakness to consider is the relatively low number of observed fetuses, especially at some GAs (after 33weeks). This could be due to a number of factors. The first and most important regards the fact that in our country the Termination Of Pregnancy can be performed up to 23 weeks of gestation. Therefore, it seems unwise to perform a measure late in gestation. The second consideration is that the diagnosis of CV anomalies must be performed after 19 weeks of gestation. However, considering the absolute number of observation after 24 weeks of gestation and stratified according to gestational age, our study continues to have the largest sample size when compared with other studies. From a statistical point of view, this observation imbalance between gestational weeks is clearly reflected by the behavior of the (approximated) confidence bands around the estimated centiles shown in Figs 7 and 8. Although rarely reported in data analyses for fetal biometry, from the perspective of good clinical practice, confidence bands are crucial in order to understand how reliable/variable the adopted reference intervals are. To take an extreme example, by looking at the 1% centile depicted in Fig 8, it seems quite nonsensical to rely on the point estimates obtained beyond 25 to 27 weeks, given the huge associated uncertainty (graphically represented by bands with a large and increasing width).

The importance of adequate reference ranges arises from the concept that evolutive lesions may affect an apparently normal CV, since vermian pathologies may develop late in pregnancy or even two years postnatally [1]. This condition leads to a difficult differential diagnosis of the various vermian abnormalities [41] and more recently a new theory has been put forward suggesting that the fact that the CV develops in a ventro dorsal direction rather than in a craniocaudal direction means that the definition of “inferior vermian hypoplasia”should be considered incorrect. Infact, both MRI an ultrasonografic studies demonstrate a linear growth of superior and inferior lobes throughout fetal life [42][43]. However, by 18 weeks the communication between the fourth ventricle and the cistern magna is covered. Therefore, the diagnosis of vermian agenesis (especially the partial form) cannot be made prior to 18 weeks’ gestation[44][45].Hence establishing the proper growth pattern of the fetal CV is important, since it has been demonstrated that the spectrum of dysgenic abnormalities of the cerebellum is expansive, ranging from subtle to important malformations.

A disturbance of the developing process of the CV may lead to complete agenesis or hypoplasia. The most common remarkable CV abnormality is the DWM which is characterized by complete agenesis or hypoplasia of the CV, dilatation of the 4^th^ ventricle and a superior displacement of the tentorium [46]. DWM is a rare brain defect which can be diagnosed both in the prenatal and neonatal period and its exact prevalence is not easy to detect(approximately 1:30.000 births) [12], since its prenatal diagnosis is difficult and its outcome could be favorable also in cases with vermian hypoplasia [47]. Moreover DWM is associated in 86% of cases with other fetal abnormalities [48] and with CNS defects in 13–67% of which the corpus callosum agenesis is the most recognized malformation[49][50].The neonatal neurological outcome of DWM is well described and the prenatal diagnosis of the complete CV agenesis form is feasible. However, the diagnosis of the DWM with a hypoplastic form or the isolated hypoplastic formis very difficult since evident signs could not be determined in the posterior fossa like in the complete form [51][52]. Viñals et al. [23] and more recently Zhao et al [53]demonstrated a reliable method to measure the height of the CV with a 3D imaging reconstruction, although comparison of 2D with 3D imaging suggests that the quality of 2D is superior and that 3D, while easy to perform, does not overcome all the limitations of 2D imaging [54].

Over the past 20 years, magnetic resonance imaging (MRI) has gained considerable importance in the evaluation of the fetal brain, usually as a complementary tool used after detection of abnormalities with ultrasound. [55]. Some authors suggest that fetal MRI studies introduce some bias in the evaluation of its sensitivity and specificity in the detection of CNS defects, concluding that ultrasound could detect CNS defects with the same accuracy especially before 25 weeks of gestation [56]. Today we can affirm that no studies with high clinical evidence have yet demonstrated the higher accuracy of MRI compared to US in the evaluation of CNS anomalies in early pregnancy.

In 2001, Malinger et al [39]provided the measurement of height of the CV in normal prenatal development, obtained transvaginally in 101 fetuses, but they provided no information regarding the statistical method used in the study. In 2004, Achiron et al[42]established reference ranges for height of fetal CV, obtained almost always by a transabdominal scan on a simple size of 292 fetuses. In 2005, Viñals et al.[23]published reference ranges for CV height and anterior-posterior diameter in 203 fetuses without providing the method utilized for the construction of the curves. In general, all of these studies have some common methodological weaknesses: a selected population, a relative low number of observed fetuses especially in some gestational ages and a no description of the statistical methods used.

In contrast to the previously published studies, we focused our attention on the statistical methodology used for the analysis of data, such as an adequate sample size (a reduced sample size will produce imprecise estimates for the reference intervals, especially on the extreme centiles), an unselected population (because reference data should relate to “normal” fetuses. Altman and Chitty [38] suggested that it is reasonable to exclude fetuses subsequently found to have a congenital abnormality, though they recommend the inclusion of neonatal deaths and fetuses large or small for dates at birth), the kind of the study (cross-sectional) and a detailed description of the statistical method (the method needs to produce reference centiles which change smoothly with gestational age and provide a good fit to the data, while maintaining, for the sake of general usability and accessibility, as simple a statistical model as possible).

## Conclusions

This is the first prospective cross-sectional study on CV height with such a large sample size and following standard statistical methods to calculate the fetal biometric reference charts. The reference interval charts we propose for fetal height of CV and corresponding reference equations have a major clinical relevance since they provide sonographers new reference equations in obstetrical practice.
